# New Board of Trade Rules

**Published:** 1917-09-22

**Authors:** 


### HOSPITALS ON EMIGRANT SHIPS.

### New Board of Trade Rules.

The Board of Trade, acting under the authority conferred on the Board by the Merchant Shipping Act, 1906, has formulated a series of new rules relative to the accommodation to be provided for steerage passengers. These rules refer to the construction of decks, to the berths required, to baths and wash-places, to lavatories, to light and ventilation, and to hospitals. The sections dealing with the last-mentioned are as follows:—

1. Spaces shall be set apart in every emigrant ship for use exclusively as hospital accommodation for the steerage passengers, and these spaces together shall contain not less than 24 superficial feet for every fifty steerage passengers carried. In no case shall a single hospital contain less than 48 superficial feet or the total hospital space contain less than 96 superficial feet. Separate and sufficient hospital accommodation shall be provided for each sex where both male and female passengers are carried.

2. The spaces set apart for hospital accommodation shall be on or above the uppermost passenger deck and shall be properly divided off from other living quarters to the satisfaction of the emigration officer and the Board of Trade medical officer at the port of construction or clearance.

3. The hospital spaces shall be fitted with bed places and supplied with proper beds, bedding and utensils, and shall be efficiently heated and ventilated to the satisfaction of the emigration officer and Board of Trade medical officer at the port of construction or clearance. There shall also be provided to the satisfaction of those officers at least one water-closet to each hospital or set of hospitals, and it shall be situated immediately adjacent to the hospitals and available for each hospital. In the case of an infectious hospital, a full-sized bath with an ample supply of hot and cold water shall be provided; the water-closet and bath may be fitted in the same space.

4. Of the hospitals provided by the above regulations at least one shall be set apart for infectious diseases, and where the number of hospitals provided is four or upwards, two of them shall be so set apart, one for males and one for females. Every hospital for infectious diseases shall be placed in as isolated a position as possible to the satisfaction of the emigration officer and the Board of Trade medical officer at the port of construction or clearance.

5. In addition to the hospital accommodation, a dispensary shall be provided, properly ventilated and fitted with shelves and drawers for medicines, dressings, instruments, and other necessaries, and a full-length couch for the examination of patients. Neither the hospitals nor the dispensary may be used for the stowage of stores, or goods of any description. The hospitals shall not be used as a sleeping apartment for other than sick steerage passengers; nor shall the dispensary be used as a sleeping place for any person.

6. In the case of new steamers, or steamers refitting or fitting for the first time as emigrant steamers, the plans of the hospitals and other accommodation shall be specially submitted to the Board of Trade principal officer at the port of construction or refitting as the case may be for consideration by the Board of Trade.

